# The effect of mepolizumab dosage form on treatment outcomes in severe asthma

**DOI:** 10.3389/fmed.2025.1537074

**Published:** 2025-04-17

**Authors:** Martina Vetchá, Kateřina Kubová, Constantinos Glynos, Sylvie Pavloková, Irena Krčmová, Eva Voláková, Ondřej Fibigr, Beáta Hutyrová, Alena Vlachová, Jiří Zeman, David Vetchý

**Affiliations:** ^1^Department of Pharmaceutical Technology, Faculty of Pharmacy, Masaryk University, Brno, Czechia; ^2^GSK Medical Department Greece, Athens, Greece; ^3^Institute of Clinical Immunology and Allergy, University Hospital, Hradec Kralove, Czechia; ^4^Faculty of Medicine in Hradec Kralove, Charles University, Hradec Kralove, Czechia; ^5^Department of Pneumology, University Hospital, Olomouc, Czechia; ^6^Faculty of Medicine and Dentistry, Palacky University, Olomouc, Czechia; ^7^Department of Pneumology, Masaryk Hospital, J.E. Purkyne University, Usti nad Labem, Czechia; ^8^Department of Pneumology and Phtiseology, University Hospital, Pilsen, Czechia; ^9^Department of Allergology and Clinical Immunology, University Hospital, Olomouc, Czechia; ^10^Department of Pneumology, Second Faculty of Medicine, Charles University and Motol University Hospital, Prague, Czechia

**Keywords:** parenteral dosage form, lyophilized injection, autoinjector, syringe, homecare, severe asthma, monoclonal antibody, mepolizumab

## Abstract

**Background:**

A monoclonal antibody such as mepolizumab typically first appears as a parenteral lyophilized formulation (LYO), then as various parenteral solution forms, and finally as a self-administered form at homecare. While more studies compare mepolizumab safety and efficacy across dosage forms, no data exists on the impact of switching to more successive dosage forms in real-world settings. This study aims to assess clinical outcomes in patients from five national Czech asthma centers who were switched from the LYO to the liquid formulation and then to home self-administration.

**Methods:**

Mepolizumab was administered in three phases: LYO for 6–9 months, followed by prefilled syringes (PFS) or autoinjectors (AI) in hospitals for 6–9 months, and finally, liquid forms at homecare for another 6–9 months. Data collected included age, BMI, nasal polyposis (NP), gastroesophageal reflux (GERD), and other comorbidities. The results were statistically evaluated using exacerbation rate (ER), asthma control test, forced expiratory volume, blood eosinophil count, and required systemic oral corticosteroid (OCS) daily dose.

**Results:**

Three months after initiation of administration, all methods showed improvement compared to the values at the beginning of treatment, with ER decreasing from a median of 4 to 0. Similarly, the median OCS decreased from 5 mg to 0 mg across all methods throughout the treatment. A more significant OCS dose reduction was observed in patients with NP (87.5% vs. 50%) and GERD (70% vs. 50%), who typically require higher OCS doses to achieve asthma control. AI/PFS outperformed LYO in ER (97.5–100% vs. 50–100% after 6–9 months of treatment) and OCS reduction (50–100% vs. 31.2–100% after 6–9 months of treatment), which was influenced rather by the later usage of AI/PFS and thus longer overall treatment times than the administrating method.

**Conclusion:**

Mepolizumab improved real-life clinical outcomes in patients with severe asthma, regardless of the dosage forms or homecare settings.

## 1 Introduction

Lyophilized formulations are often the first choice for the dosage form of protein drugs because they improve the formulation stability based on a general phenomenon of reduced molecular mobility and degradation kinetics in the dried state (1). Liquid dosage forms can be in the form of prefilled syringes or more sophisticated autoinjectors. Patients preferred the autoinjector device for self-administration at homecare, rating it as the easiest and most intuitive (2). Although liquid dosage forms are the most preferred due to the highest level of clinician and patient compliance, mainly because of the elimination of the reconstitution step and possibly self-administration at homecare, they exhibit a variety of physical and chemical forms of degradation. Chemical degradation refers to modifications involving covalent bonds, such as deamidation, oxidation, and disulfide bond shuffling. Physical degradation includes protein unfolding, undesirable adsorption to surfaces, and aggregation (3).

In addition, high-concentration liquid formulations are often required for the subcutaneous delivery of monoclonal antibody formulations. Issues such as induced viscosity, phase separation, opalescence, or self-association can be observed from molecular crowding effects (1, 4). Liquid dosage forms are therefore more difficult to produce, require a greater need for cold-chain storage, and have more stringent transportation criteria. For these reasons, liquid dosage forms have appeared on the market mainly after the introduction of lyophilized formulations.

In 2015, the European Commission granted marketing authorization for Nucala^®^ (mepolizumab) as a lyophilized formulation (LYO). The efficacy and safety of mepolizumab in patients with severe eosinophilic asthma in randomized controlled trials have been well established (5–8). The REALITI-A study demonstrated that real-world treatment with mepolizumab was clinically effective in patients with severe asthma, providing disease control while reducing both exacerbation rate and the need to maintain oral corticosteroid use (9).

From 2019, the new dosage forms of a prefilled syringe (PFS) and a prefilled pen (autoinjector, AI) are available on the European market with similar pharmacokinetic properties to the lyophilized formulation and no identified additional safety concerns (10). Moreover, patients/caregivers have successfully self-administered mepolizumab via the autoinjector or the prefilled safety syringe both in the clinic environment as well as at home (11, 12).

As the real-world use of mepolizumab has increased, more data on its use have appeared in the scientific literature. Data on mepolizumab are now available relating to a broad range of clinical outcomes, safety, and healthcare resource use (9, 13). However, the use of mepolizumab has not yet been evaluated in patients who have switched to another dosage form and homecare setting treatment. The aim of this retrospective analysis was to assess the mepolizumab treatment outcomes in patients who were switched from the lyophilized formulation to the liquid formulation and then to home self-administration.

## 2 Materials and methods

### 2.1 Study design

This retrospective analysis included data from five national centers for the treatment of severe asthma in the Czech Republic. The effect of the mepolizumab administration method on treatment outcomes in patients with severe asthma was evaluated. Patients included in the assessment were treated with lyophilized formulation for 6–9 months, then followed by 6–9 months of treatment with liquid forms administered in a hospital setting as they became available in the Czech Republic, and finally switched to homecare and evaluated for 6–9 months from 2019 to 2022. All included patients were biological-naïve. The switch to another form of drug administration was conditioned by patient agreement. The project was approved by the Ethics Committee of Masaryk University (EKV-2024-059) and the Motol University Hospital (EK – 21/24).

The study design reflects the real-world use of mepolizumab dosage forms in the treatment of severe asthma. At the first visit to a national center for severe asthma treatment, patients were put on a lyophilized formulation of mepolizumab. After 3 months of treatment, they were usually checked on their second visit. At follow-up, after 6–9 months of treatment with the lyophilized form, patients were switched to the liquid form of mepolizumab. They were followed up after 3 months and again, usually after 6–9 months, were switched to homecare if they had been assessed as responders after 12 months of treatment.

All patients had to meet the reimbursement criteria for mepolizumab treatment in the Czech Republic, which were either four severe asthma exacerbations in the 12 months prior to initiation of mepolizumab therapy and a blood eosinophil count above 300 cells/μL, or the need for at least 6 months of maintenance treatment with oral corticosteroids (OCS, equivalent to 5 mg prednisolone) and blood eosinophil count above 300 cells/μL 12 months before the OCS initiation. According to Czech reimbursement criteria, patients are assessed as responders after 12 months of treatment. A 50% reduction in exacerbation rate or a significant reduction in daily OCS dose must be achieved.

Exclusion criteria for the analysis included pre-specified concurrent medical conditions such as another respiratory disease, current eosinophilic disease other than severe eosinophilic asthma, known and pre-existing parasitic infection within 6 months of screening, active smoking, use of prohibited concomitant medications, history of alcohol/substance abuse, or hypersensitivity to any component of the study medication.

### 2.2 Data collection

Anonymous patient data were collected from five national centers. The data included patient characteristics and outcomes of their mepolizumab treatment at the time of initiation, after 3 months of treatment with the lyophilized formulation, after 6–9 months of treatment with the lyophilized formulation, after 3 months of treatment with the liquid formulation, after 6–9 months of treatment with the liquid formulation, after 3 months of homecare, and after 6–9 months of homecare.

The patient’s age, body mass index (BMI), presence of nasal polyposis (NP), gastroesophageal reflux (GERD), and other comorbidities were recorded. The diagnosis of GERD, as well as other comorbidities, was based on the hospital registry information. The mepolizumab treatment outcomes with different administration methods were assessed by using the values of blood eosinophil count (BEC), exacerbation rate (ER), asthma control test (ACT), forced expiratory volume (FEV1), and the daily dose of systemic oral corticotherapy required to maintain asthma control (OCS).

### 2.3 Statistical analysis

The input dataset contained five measured variables (BEC, ER, ACT, FEV1, OCS) for 66 patients at three time points (0, 3, and 6–9 months) for three administration methods (LYO, AI/PFS, homecare). For better comparability of treatment response among patient groups, the absolute values of each quantity were also recalculated to the relative values. This was done by determining the rate of quantity change over time compared to the original value (i.e., 3 vs. 0 months and 6–9 vs. 0 months). The resulting relative value was calculated as the percentage improvement. For variables where an increase over time is desired (ACT, FEV1), it was the growth rate; for variables where a decrease over time is desired (BEC, ER, OCS), it was the rate of decrease. Therefore, a higher relative value (%) indicates a better response to treatment. Statistical evaluation was performed for relative or absolute values, depending on the type of test and the purpose of the output and interpretation.

In the input dataset, values of not quite all quantities (out of 5) were available for each patient at each sampling point. The output statistics and various tests are based only on the available values. This fact is due to the common clinical practice in each particular center as this assessment fully reflects the standard practice in Czech severe asthma centers, e.g., if there is no exacerbation present and the ACT is above 20, the BEC is usually not assessed, and spirometry (FEV1) is usually performed every 6 months.

The non-normality of the data in all tested subgroups was confirmed by the Shapiro–Wilk test. Therefore, non-parametric statistical approaches were applied for subsequent data analysis. Data visualization was performed using a box and whisker plot showing the median (a middle line dividing the box), IQR (a box), minimum/maximum score (whiskers), and possible outliers. Descriptive statistics based on robust parameters – median and interquartile range (IQR) – were used to summarize all quantities across groups and subgroups of the entire dataset. The Wilcoxon test for paired data was used to compare the values of individual variables between each two consecutive sampling points. In our case, it was used to compare the values of individual monitored variables for individual patients over time. The Mann–Whitney U test (for two subgroups) or the Kruskal-Wallis test (for three subgroups) followed by Dunn’s multiple comparison test was used to compare the values of individual variables between different data subsets at different time points. These tests were used as an extension of the Wilcoxon test assessment to consider different values of each quantity for individual patients at time 0 months. The tests were used to determine whether the rate of change of a given quantity in a given time period is comparable across groups. The determination of which group of patients has a higher/lower rate of improvement is derived from a comparison of specific data in Supplementary Table 1.

The non-parametric correlation coefficient, Spearman’s rho (r_s_), was used to assess the association between the values of each quantity and age. For correlation analysis, age was treated as a continuous variable; in other cases, age was converted into subgroups (< 45, 45–55, 55–65, and > 65 years) to achieve simple data segmentation with an even distribution of patients in the age subgroups and at the same time for a sufficient age difference between younger and older patients.

Analysis was performed on the entire dataset, individual groups, and subgroups. Stratification was performed based on the mepolizumab administration method, age, BMI, NP, and GERD, and then for combinations of the mepolizumab administration method and each level of all other parameters. The effects were investigated for the most represented comorbidities (NP, GERD); others could not be assessed accurately due to the low number of cases.

R software version 4.1.2 was used for data analysis (14).

## 3 Results

### 3.1 Patient characteristics and basic descriptive statistics

A total of 66 patients met the study criteria and were included in the evaluation (Table 1). The mean age was 55.0 years, and the majority (62.1%) was aged between 45 and 65 years. Healthy weight, overweight, and obese patients were equally represented in the study. The most common comorbidities included GERD (62.1%) and NP (42.4%) followed by allergic rhinitis (15.2%) and diagnosed immunodeficiency (10.6%). The treatment response rate in the study was 87.9%. These patients experienced a 50% or greater reduction in the number of exacerbations per year or a significant reduction in the dose of OCS during treatment, as defined by reimbursement criteria.

**Table 1:** Patient characteristics and basic descriptive statistics.

Patients, n	66
Age (years)	
Mean (SD)	55.0 (10.8)
Range	21–75
Age group (years), n (%)	
<45	12 (18.2)
45–55	14 (21.2)
55–65	27 (40.9)
> 65	13 (19.7)
BMI, n (%)	
< 25	29 (43.9)
25–30	16 (24.2)
> 30	21 (31.8)
NP, n (%)	
Yes	28 (42.4)
No	37 (56.1)
Not available value	1 (1.5)
GERD, n (%)	
Yes	41 (62.1)
No	24 (36.4)
Not available value	1 (1.5)
Other comorbidities, n (%)	
Allergic rhinitis	10 (15.2)
Diagnosed immunodeficiency	7 (10.6)
Aspirin sensitivity AERD	3 (4.5)
Atopic dermatitis	2 (3.0)
Urticaria	1 (1.5)
Eosinophilic pneumonia	1 (1.5)
Depression	1 (1.5)
IgE levels above 700 IU/mL	1 (1.5)
Allergic bronchopulmonary aspergillosis	1 (1.5)
Obstructive sleep apnoea	1 (1.5)
Vocal cord dysfunction	1 (1.5)
Responder	
Responder	58 (87.9)
Non-responder	8 (12.1)

Descriptive statistics of the percentage rate of improvement of each monitored outcome at a given time (3 and 6–9 months) compared to time 0 are listed in Supplementary Table 1.

### 3.2 Evaluation of changes in monitored quantities over time

**Figure 1:**
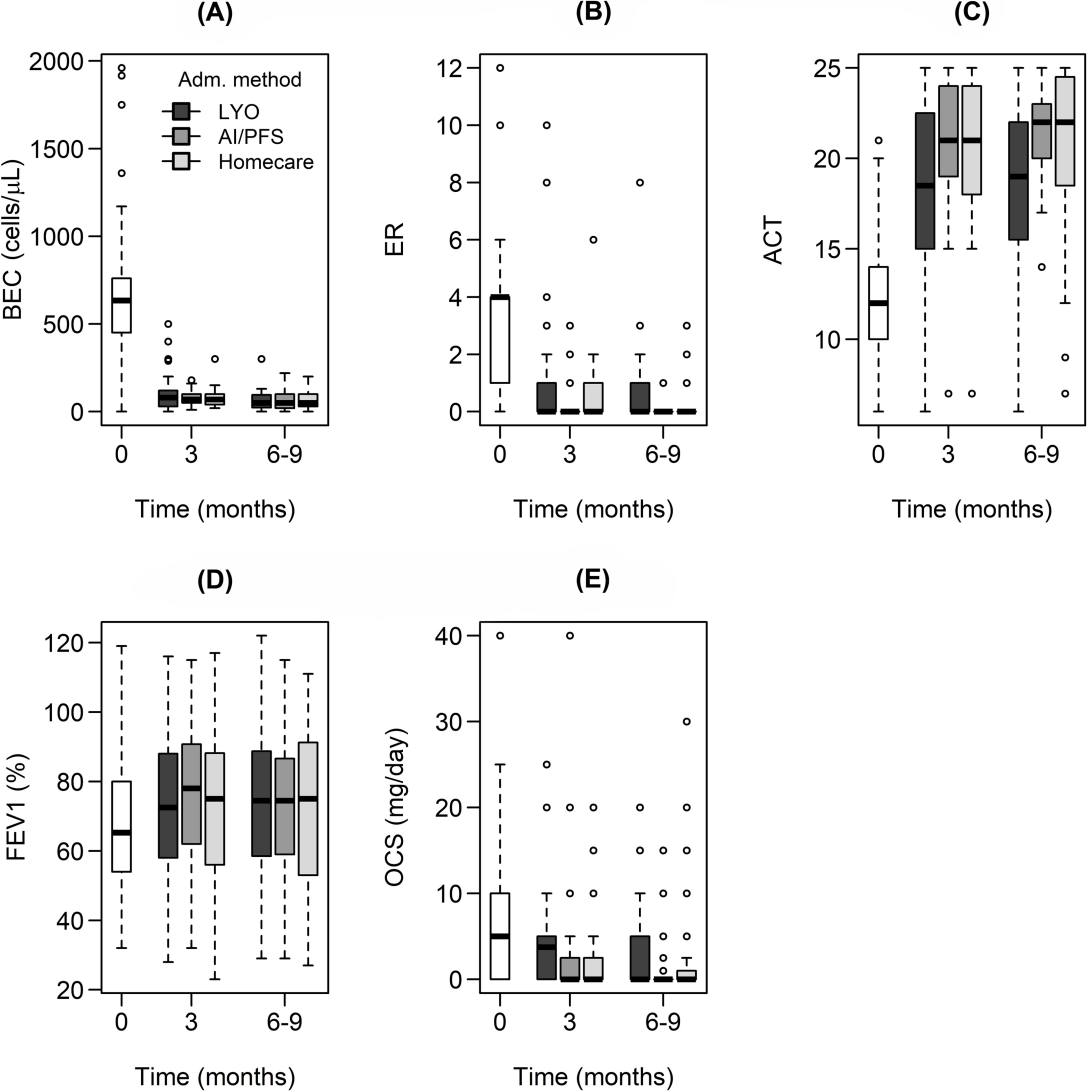
Box and whisker plots showing absolute values of the monitored outcomes [**(A)** blood eosinophil count, **(B)** exacerbation rate, **(C)** asthma control test, **(D)** forced expiratory volume, **(E)** daily dose of systemic oral corticotherapy required to maintain asthma control] depending on time for individual data groups stratified by the mepolizumab administration method and differentiated by box color. A statistically significant difference in the monitored variables between 3 and 0 months was confirmed for all cases and all administration methods. On the contrary, there was no significant change when comparing 6–9 months to 3 months except for BEC in patients treated with LYO or PFS/AI and OCS in patients treated with LYO.

The Wilcoxon paired test confirmed a statistically significant improvement in all monitored variables after 3 months of treatment (i.e., the decrease in BEC, ER, and OCS and the increase in ACT and FEV1), as shown in Figure 1 and Supplementary Table 2, in almost all cases, regardless of the mepolizumab administration method and patient characteristics (age, BMI, and the two most represented comorbidities – NP and GERD). There was no significant improvement only in patients over 65 years for FEV1.

After 6–9 months of treatment, no further statistically significant changes in ER, ACT, and FEV1 were observed. On the contrary, an additional decrease in BEC was observed in the following groups: patients treated with LYO or PFS/AI, patients aged 45–65 years, and patients with a BMI below 25 or above 30. A decrease in BEC was observed regardless of the presence of NP or GERD. Additional reductions in OCS were observed in the groups of patients treated with LYO, patients aged 45–55 years or over 65 years, patients with a BMI less than 30, and again independently of the presence of NP or GERD (Supplementary Table 1; Figure 1).

For all considered subgroups, a reduction in BEC of more than 80% was observed after 3 months of treatment, and it remained at similar values even after 6–9 months (Supplementary Table 2; Figure 1A). Decreases in ER and OCS were greater than 95 and 50%, respectively, for all administration methods throughout the treatment period. In terms of absolute values, ER dropped from a baseline median of 4 to a median of 0. Similarly, the median of OCS 5 at time 0 months decreased to a value of 0 for all methods of mepolizumab administration throughout the treatment period (Table 2; Figures 1B,E; Supplementary Table 1).

**Table 2:** Effect of the mepolizumab administration method: descriptive statistics in “median (IQR), n” format of monitored outcomes at 0 and 6–9 months.

Time	Mepolizumab administration method	BEC (cells/μL)	ER (exacerbation number/year)	ACT	FEV1 (%)	OCS (mg of prednisolone/day)
0 months	–	634.0 (458.0–755.0), 66	4.0 (1.0–4.0), 65	12.0 (10.0–14.0), 46	65.2 (54.2–80.0), 66	5.0 (0.0–10.0), 66
6–9 months	LYO	50.0 (23.5–95.0), 39	0.0 (0.0–1.0), 53	19.0 (15.5–22.0), 39	74.5 (59.2–88.6), 52	0.0 (0.0–5.0), 55
	AI/PFS	50.0 (20.0–100.0), 29	0.0 (0.0–0.0), 37	22.0 (20.0–23.0), 25	74.5 (60.0–85.8), 36	0.0 (0.0–0.0), 41
	Homecare	50.0 (30.0–100.0), 32	0.0 (0.0–0.0), 49	22.0 (18.5–24.5), 35	75.0 (53.8–90.2), 42	0.0 (0.0–1.0), 49

The starting condition (reimbursement criteria) effect on FEV1 at 3 months was also examined (Table 3). In patients with exacerbations as a reason for mepolizumab initiation, FEV1 increased for all mepolizumab administration methods. FEV1 increased significantly only in patients with PFS/AI when the reason for mepolizumab initiation was maintenance OCS treatment.

**Table 3:** Effect of time: differences in FEV1 between 6–9 and 3 months for the selected groups stratified by the presence of ER and OCS at the beginning of treatment.

Patients	Mepolizumab administration method		
	LYO	PFS/AI	Homecare
All with ER	**Increase (*p* = 0.001)**	**Increase (*p* = 0.003)**	**Increase (*p* = 0.015)**
With ER and without OCS	**Increase (*p* = 0.004)**	**Increase (*p* = 0.002)**	**Increase (*p* = 0.021)**
All with OCS	NS (*p* = 0.070)	**Increase (*p* = 0.011)**	NS (*p* = 0.233)
With OCS and without ER	NS (*p* = 0.317)	**Increase (*p* = 0.012)**	NS (*p* = 0.239)
With ER and OCS	**Increase (*p* = 0.041)**	NS (*p* = 0.483)	NS (*p* = 0.625)

The results are in the format: an increase/decrease in FEV1 over time or a statistically insignificant difference with the corresponding p-value from the Wilcoxon paired test (significant differences are indicated in bold). NS means “not significant difference.”

Only the AI/PFS route was associated with an increase in FEV1 in patients receiving corticosteroids. On the other hand, only the LYO administration method increased FEV1 in patients suffering from exacerbations at the beginning of treatment and taking corticosteroids.

### 3.3 The effect of the mepolizumab administration method and patient characteristics on the rate of improvement in monitored quantities

The results of the Mann–Whitney U test and the Kruskal-Wallis test are summarized in Table 4. The effect of age and BMI on the rate of improvement of the patients’ condition was not found to be statistically significant for almost any of the parameters monitored at any time point. On the contrary, it was confirmed that patients with NP and GERD had a higher rate of improvement in some measures compared to patients without these comorbidities (Supplementary Table 1).

**Table 4:** Effect of the mepolizumab administration method and basic patient characteristics: differences in the monitored quantities between 3 and 0 months, and 6–9 and 0 months for the entire dataset, and the effect of the mepolizumab administration method for selected groups stratified by age, BMI, NP, and GERD.

Quantity	Data		3 vs. 0 months					6–9 vs. 0 months				
	Grouping variable	Group	BEC	ER	ACT	FEV1	OCS	BEC	ER	ACT	FEV1	OCS
Mepolizumab administration method	–	Entire data set	0.385	**0.016**	0.222	0.418	**< 0.001**	0.998	**< 0.001**	0.421	0.802	**0.017**
	Age (years)	< 45	0.424	0.233	0.253	0.997	**0.036**	0.355	0.238	0.402	0.281	0.223
		45–55	0.715	**0.002**	0.640	0.707	**0.005**	0.193	**0.015**	0.331	0.816	0.115
		55–65	0.846	0.868	0.380	0.857	**0.018**	0.436	0.183	0.715	0.619	0.413
		> 65	0.347	0.342	0.711	0.138	0.189	0.630	0.102	0.674	0.294	0.359
	BMI	< 25	0.354	0.177	0.620	0.496	**< 0.001**	0.771	**0.006**	0.260	0.776	**0.035**
		25–30	0.986	0.846	0.672	0.275	**0.017**	0.891	0.419	0.949	0.761	0.275
		> 30	0.902	**0.030**	0.510	0.958	0.321	0.552	0.136	0.694	0.957	0.412
	NP	Yes	0.393	0.236	0.264	0.566	**0.002**	0.979	0.113	0.390	0.853	**0.050**
		No	0.436	**0.021**	0.295	0.464	**0.006**	0.928	**0.002**	0.187	0.883	0.313
	GERD	Yes	0.382	0.749	0.471	0.546	**0.001**	0.979	0.081	0.648	0.817	0.085
		No	0.796	**< 0.001**	0.591	0.737	**0.015**	0.827	**0.003**	0.072	0.194	0.228
Age	–	Entire data set	0.369	0.204	**0.012**	0.089	0.887	0.481	0.989	0.083	0.558	0.492
BMI			0.483	0.088	0.091	0.379	0.097	0.194	0.428	0.235	0.186	**0.014**
NP			0.536	**0.007**	0.877	0.291	**0.034**	**0.006**	**< 0.001**	0.162	0.142	**0.009**
GERD			**0.002**	**0.005**	**0.029**	0.408	0.182	**< 0.001**	0.158	**< 0.001**	0.745	0.667

The results are presented as *p*-values of Mann–Whitney U test in case of two subgroups or Kruskal-Wallis test for three and four subgroups (significant effects are indicated in bold).

In terms of the rate of improvement in BEC, ACT, and FEV1 at both sampling points after treatment, all types of treatment were evaluated as equivalent. In contrast, AI/PFS and home treatment were associated with a higher rate of exacerbation and OCS improvement than LYO, which was influenced rather by the later usage of AI/PFS and thus longer overall treatment times than the administrating method.

### 3.4 Correlation analysis

The correlation analysis is presented in Table 5. It can be concluded that the age of the patients did not have a statistically significant relationship with the rate of improvement of the patients’ condition for the entire dataset, as well as for individual groups according to the mepolizumab administration method.

**Table 5:** Correlation analysis: correlation between the monitored quantities and age for the entire dataset and individual groups stratified by the mepolizumab administration method.

Time (months)	Quantity	Entire data set	Data group		
			LYO	AI/PFS	Homecare
3	BEC	0.12 (0.241)	0.15 (0.318)	0.00 (0.982)	0.12 (0.514)
	ER	−0.09 (0.323)	0.04 (0.812)	−0.08 (0.608)	**−0.31 (0.049)**
	ACT	**0.34 (0.001)**	**0.42 (0.013)**	0.35 (0.083)	0.28 (0.147)
	FEV1	0.03 (0.710)	−0.06 (0.663)	0.16 (0.297)	0.03 (0.823)
	OCS	0.00 (0.969)	0.19 (0.231)	−0.17 (0.360)	−0.02 (0.924)
6–9	BEC	−0.09 (0.395)	−0.26 (0.110)	−0.11 (0.570)	0.17 (0.351)
	ER	0.02 (0.818)	0.03 (0.827)	0.17 (0.373)	−0.09 (0.584)
	ACT	**0.26 (0.019)**	**0.37 (0.035)**	0.19 (0.425)	0.21 (0.283)
	FEV1	0.10 (0.258)	0.11 (0.456)	0.24 (0.158)	−0.03 (0.862)
	OCS	0.10 (0.343)	0.19 (0.254)	−0.02 (0.911)	0.08 (0.642)

The results are presented in Spearman’s rho format with the corresponding p-value in parenthesis, indicating the statistical significance of the correlation (significant r_s_ values are shown in bold).

### 3.5 Safety evaluation

During the assessed period, 11 mepolizumab-related adverse events (AEs) occurred in seven patients (11%) of the 66 patients included in the analysis. The most common AEs were asthma exacerbations (6) and infections (3, including one COVID-19). One death not related to the treatment was reported. Eight out of 66 patients (12.1%) had discontinued mepolizumab after lyophilized formulation treatment due to inadequate control (*n* = 7) and atopic dermatitis worsening (*n* = 1, the patient was switched to dupilumab).

## 4 Discussion

The aim of this retrospective analysis was to assess the effect of the mepolizumab dosage forms on the treatment outcomes in the real-world use of mepolizumab in the Czech Republic. Patients from five national centers for the treatment of severe asthma took lyophilized formulations for 6–9 months, followed by 6–9 months of liquid forms administered in a hospital, and then 6–9 months of liquid forms in a homecare setting. Before the COVID-19 pandemic, patients in severe asthma clinics were mainly treated with liquid forms. Treatment adherence in asthma is variable and depends on many factors, such as acceptance of the disease, relationship and communication with healthcare professionals, education of patients, and their attitudes and beliefs (15). Epidemiological restrictions in the COVID-19 pandemic rapidly accelerated the spread of self-administration. The use of homecare is now standard in the Czech Republic for the management of severe asthma. According to the latest validation, 73% of patients receiving mepolizumab are treated in a homecare setting. Homecare is slightly more common for biologics with more frequent dosing intervals (86% for both omalizumab and dupilumab) than for those with less frequent dosing intervals (70% for benralizumab) (16). However, there is very limited data available on how the transition to self-administration in the homecare setting affects clinical outcomes in severe asthma.

### 4.1 Patient characteristics and basic descriptive statistics

The patients included in this analysis, which reflects real-world use in the Czech Republic, differed from those in randomized controlled trials (RCTs) such as the MUSCA, MENSA, or DREAM study (6–8). The enrolled patients were approximately 5 years older, and more patients had nasal polyposis (42%) than did patients in MENSA (16%), MUSCA (21%), and DREAM (7–14%). However, this proportion was similar to that observed in other real-world (RWE) studies of patients with severe asthma (39, 46%) (9, 13). On the other hand, the second observed comorbidity, GERD, was significantly more common in patients in this analysis (62%) than in published real-world studies (38, 21%) (9, 13), but in line with an estimated prevalence of GERD in the severe asthma population (17). In another Czech published cohort, GERD was similarly common (64.7%) (18). The difference in patient demographics between this analysis and the MENSA, MUSCA, and DREAM RCTs, apart from the imposition of strict inclusion and exclusion criteria in the RCTs, reflects the Czech regulatory reimbursement criteria for mepolizumab treatment following its approval. In the Czech Republic, only patients with a BEC greater than 300 cells/μL and at least four exacerbations in the previous 12 months, or 6 months of OCS maintenance treatment could have been initiated on mepolizumab. These criteria are stricter than those for RCTs. Patients were started at later stages of the disease, usually with more pronounced and advanced comorbidities. Also, using a 300-cell cut-off enables selection of patients with a more eosinophilic disease phenotype where comorbidities such as nasal polyposis are more frequent (19, 20). Later treatment initiation may also explain the higher frequency of GERD in our cohort (17). Higher cumulative doses of OCS or maintenance treatment with OCS may increase the risk of developing GERD (21). A patient was considered a responder if they achieved at least a 50% reduction in ER or a significant reduction in OCS dose. Assessment was required after every 12 months of treatment. Accurate phenotyping and treatment of comorbidities in the Czech Republic resulted in a high response rate to treatment.

### 4.2 Evaluation of changes in monitored quantities over time

After 3 months of treatment, there was an overall improvement in all monitored outcomes across all dosing regimens (Supplementary Table 2; Figure 1). The ER reached the almost ideal target value of 0. Similarly, the desired reduction in OCS was achieved and maintained regardless of the administration method and the presence of comorbidities, with no impact on the improvement in ACT, which was also seen across all methods of administration. From the clinical point of view, it was considered an excellent treatment response. The monitored levels of ER and ACT reached their maximum potential for improvement from a statistical point of view. Only FEV1 in patients over 65 years of age showed a non-significant improvement, which could have been due to fixed obstruction, as is common in older patients with limited potential for improvement.

A slightly higher improvement in ER and OCS dose reduction was observed in AI/PFS than in LYO throughout the entire observation period, thus confirming further improvement in disease control over time without the impact of the change in the administration method (Supplementary Table 1). A significant and sustained decrease in ER is fully consistent with mepolizumab RCTs (6–8) and the RWE studies REALITI-A and REDES (9, 13). A reduction in the median from 4 to 0 was sustained for all dosage forms and achieved independently due to age, BMI, or the presence of NP and GERD (Table 2). An additional decrease in BEC after 6–9 months was observed in the following groups: patients treated with LYO or PFS/AI, patients aged 45–65 years, and those with BMI below 25 or above 30. However, the reduction was small with no clinical impact, confirming adequate disease control. As the reduction rate in BEC was observed independently of the presence of NP or GERD, it could be confirmed that there was no comorbidity influence on this biomarker.

Additional reductions in OCS after 6–9 months were seen in the LYO-treated groups, in patients aged 45–55 years or over 65 years, in patients with a BMI of less than 30, and again regardless of the presence of NP or GERD (Supplementary Table 2; Figure 1). The differences observed are fully consistent with clinical practice. Tapering of the OCS dose was started at the very beginning of the treatment (LYO). The pace of the OCS dose tapering was individualized, led by asthma control, and adjusted to the baseline dose. In the later stages of the treatment (AI/PFS and homecare), the OCS dose median was 0, therefore there were no further changes. A similar reduction in ER (mean from 4.4 to 0.7 after 12 months) and OCS dose (mean from 11.8 to 3 mg) was described in a smaller RWE cohort from the Czech Republic (18). The results of another retrospective study from Slovakia were comparable; the median ER decreased from 5 to 0, and the median OCS dose decreased from 15 to 6.25 mg after 12 months. In this cohort, 53% of patients had a BMI above 30, and 82% were OCS dependent (22).

The difference between the increase in FEV1 in patients who were initiated on the basis of at least four exacerbations and those on OCS maintenance (Table 3) could be explained by earlier initiation of the treatment in exacerbating patients and better-preserved lung functions compared to patients requiring OCS where the FEV1 improvement is milder and was achieved later in patients on AI/PFS. Moreover, OCS tapering affected FEV1 in OCS-dependent patients at the beginning of the treatment. Switching to homecare did not affect monitored quantities.

### 4.3 The effect of the mepolizumab administration method and patient characteristics on the monitored outcomes

More pronounced OCS dose reduction was observed in patients with NP and GERD, as patients with comorbidities tend to have a more severe disease, requiring higher OCS doses to achieve asthma control. Mepolizumab improved outcomes regardless of the presence of comorbidities across all administration methods. The same outcomes (irrespective of comorbidity presence) were also observed in the REALITI-A study, its sub-analysis, and the sub-analysis of RCTs (23–26). A higher rate of exacerbations and OCS improvement for AI/PFS and homecare than for lyophilizate showed that the full effect of mepolizumab could usually be achieved after several months of the treatment.

### 4.4 Correlation analysis

No clinically relevant correlation of patients’ age with treatment outcomes was observed in the cohort of patients studied. Safety outcomes were consistent with those seen in the RCTs and other RWE sites.

In conclusion, this retrospective analysis showed that mepolizumab improved real-life clinical outcomes in patients with severe asthma in five severe asthma centers in the Czech Republic, irrespective of different dosage forms or homecare settings, confirming the minimal influence of factors connected with compliance and other risks associated with the place of administration, the person administering the dose, training, and experience. It could also be concluded that there was no significant influence of age, BMI, or monitored comorbidity on treatment outcomes observed in the patients studied. These findings are consistent with the results from clinical trials showing that mepolizumab reduces the ER and OCS dose rate across a range of clinical characteristics and comorbidities and administration methods in clinical practice.

The study has some limitations. The study’s conclusions are limited by the limited number of patients included in the analysis. This limitation must be particularly considered in the case of the influence of mepolizumab administration methods for groups stratified by age, BMI, NP, and GERD shown in Table 4.

The order of mepolizumab administration methods is the same in all patients, so the impact of changing a different order is not evaluated.

## Data Availability

The data analyzed in this study is subject to the following licenses/restrictions: the dataset will be available on request. Requests to access these datasets should be directed to kubovak@pharm.muni.cz.
