# Supplementary material for: The effect of mepolizumab dosage form on treatment outcomes in severe asthma
Source: Front Med (Lausanne). 2025 Apr 17;12:1537074. doi: 10.3389/fmed.2025.1537074 (PMC12054249; doi:10.3389/fmed.2025.1537074)
Supplement: Supplementary file 1 [file Data_Sheet_1.pdf]

Supplementary Material

Supplementary Table 1. Effect of time: differences in the monitored quantities between 3 and 0 months, and 6–9 and 3 months for the selected groups stratified by the mepolizumab administration method, age, BMI, NP, and GERD. The results are presented as an increase/decrease in quantity over time or a statistically insignificant difference with the corresponding p-value from the Wilcoxon paired test (significant differences are indicated in bold).

**Quantity**	**Group**	**3 vs. 0 months**				
		**BEC**	**ER**	**ACT**	**FEV1**	**OCS**
Mepolizumab administration method	LYO	**Decrease (p < 0.001)**	**Decrease (p < 0.001)**	**Increase (p < 0.001)**	**Increase (p < 0.001)**	**Decrease (p < 0.001)**
	AI/PFS	**Decrease (p < 0.001)**	**Decrease (p < 0.001)**	**Increase (p < 0.001)**	**Increase (p < 0.001)**	**Decrease (p < 0.001)**
	Homecare	**Decrease (p < 0.001)**	**Decrease (p < 0.001)**	**Increase (p < 0.001)**	**Increase (p = 0.014)**	**Decrease (p < 0.001)**
Age (years)	< 45	**Decrease (p < 0.001)**	**Decrease (p < 0.001)**	**Increase (p < 0.001)**	**Increase (p = 0.033)**	**Decrease (p = 0.004)**
	45–55	**Decrease (p < 0.001)**	**Decrease (p < 0.001)**	**Increase (p < 0.001)**	**Increase (p = 0.006)**	**Decrease (p < 0.001)**
	55–65	**Decrease (p < 0.001)**	**Decrease (p < 0.001)**	**Increase (p < 0.001)**	**Increase (p < 0.001)**	**Decrease (p < 0.001)**
	> 65	**Decrease (p < 0.001)**	**Decrease (p < 0.001)**	**Increase (p < 0.001)**	NS (p = 0.084)	**Decrease (p = 0.004)**
BMI	< 25	**Decrease (p < 0.001)**	**Decrease (p < 0.001)**	**Increase (p < 0.001)**	**Increase (p < 0.001)**	**Decrease (p < 0.001)**
	25–30	**Decrease (p < 0.001)**	**Decrease (p < 0.001)**	**Increase (p = 0.002)**	**Increase (p < 0.001)**	**Decrease (p < 0.001)**
	> 30	**Decrease (p < 0.001)**	**Decrease (p < 0.001)**	**Increase (p < 0.001)**	**Increase (p = 0.002)**	**Decrease (p = 0.007)**
NP	Yes	**Decrease (p < 0.001)**	**Decrease ((p < 0.001)**	**Increase (p < 0.001)**	**Increase (p < 0.001)**	**Decrease (p < 0.001)**
	No	**Decrease (p < 0.001)**	**Decrease (p < 0.001)**	**Increase (p < 0.001)**	**Increase (p = 0.035)**	**Decrease (p < 0.001)**
GERD	Yes	**Decrease (p < 0.001)**	**Decrease (p < 0.001)**	**Increase (p < 0.001)**	**Increase (p < 0.001)**	**Decrease (p < 0.001)**
	No	**Decrease (p < 0.001)**	**Decrease (p < 0.001)**	**Increase (p < 0.001)**	**Increase (p < 0.001)**	**Decrease (p < 0.001)**
**Quantity**	**Group**	**6–9 vs. 3 months**				
		**BEC**	**ER**	**ACT**	**FEV1**	**OCS**
Mepolizumab administration method	LYO	**Decrease (p < 0.001)**	NS (p = 0.754)	NS (p = 0.691)	NS (p = 0.977)	**Decrease (p < 0.001)**
	AI/PFS	**Decrease (p = 0.017)**	NS (p = 0.777)	NS (p = 0.260)	NS (p = 0.183)	NS (p = 0.186)
	Homecare	NS (p = 0.466)	NS (p = 0.275)	NS (p = 0.514)	NS (p = 0.055)	NS (p = 0.106)
Age (years)	< 45	NS (p = 0.050)	NS (p = 0.454)	NS (p = 0.102)	NS (p = 0.316)	NS (p = 0.076)
	45–55	**Decrease (p = 0.012)**	NS (p = 0.821)	NS (p = 0.944)	NS (p = 0.864)	**Decrease (p = 0.034)**
	55–65	**Decrease (p = 0.042)**	NS (p = 0.437)	NS (p = 1.000)	NS (p = 0.894)	NS (p = 0.075)

	> 65	NS (p = 0.090)	NS (p = 0.588)	NS (p = 0.502)	NS (p = 0.227)	**Decrease (p = 0.020)**
BMI	< 25	**Decrease (p = 0.003)**	NS (p = 0.847)	NS (p = 0.387)	NS (p = 0.149)	**Decrease (p = 0.015)**
	25–30	NS (p = 0.255)	NS (p = 0.233)	NS (p = 0.591)	NS (p = 0.075)	**Decrease (p = 0.002)**
	> 30	**Decrease (p = 0.002)**	NS (p = 0.548)	NS (p = 0.570)	NS (p = 0.059)	NS (p = 0.096)
NP	Yes	**Decrease (p = 0.004)**	NS (p = 0.694)	NS (p = 0.098)	NS (p = 0.220)	**Decrease (p = 0.004)**
	No	**Decrease (p = 0.003)**	NS (p = 0.851)	NS (p = 0.939)	NS (p = 0.670)	**Decrease (p = 0.002)**
GERD	Yes	**Decrease (p = 0.001)**	NS (p = 0.560)	NS (p = 0.096)	NS (p = 0.793)	**Decrease (p = 0.003)**
	No	**Decrease (p = 0.021)**	NS (p = 0.394)	NS (p = 0.659)	NS (p = 0.410)	**Decrease (p = 0.003)**

Abbreviations: ACT, asthma control test; AI, autoinjectors; BEC, blood eosinophil count; BMI, body mass index; ER, exacerbation rate; FEV1, forced expiratory volume; GERD, gastroesophageal reflux; LYO, lyophilised formulation; NP, nasal polyposis; NS, not significant difference; OCS, oral corticosteroids; PFS, prefilled syringes

Supplementary Table 2. Descriptive statistics in the format "median (IQR), n" of monitored quantities at both sampling points for selected groups and subgroups stratified by mepolizumab administration method, age, BMI, NP, and GERD. Quantities are expressed as the percentage rate of improvement of a given quantity at a given time compared to time zero; thus, a higher value indicates a better response to treatment.

**Variable**	**Group**	**Subgroup**	**The rate of improvement of a given quantity (3 vs. 0 months) (%)**				
			**BEC**	**ER**	**ACT**	**FEV1**	**OCS**
Mepolizumab administration method	LYO	–	88.0 (82.2–93.5), 44	100.0 (50.0–100.0), 48	40.0 (17.2–87.5), 35	9.5 (−2.9–22.4), 58	50.0 (0.0–50.0), 42
	AI/PFS	–	89.9 (86.2–92.9), 30	100.0 (100.0–100.0), 40	55.2 (27.1–104.0), 26	15.3 (5.7–27.2), 44	100.0 (50.0–100.0), 32
	Homecare	–	92.3 (85.2–94.1), 31	100.0 (80.8–100.0), 42	53.3 (33.3–118.0), 29	10.8 (−2.0–28.1), 47	100.0 (50.0–100.0), 33
Age (years)	< 45	–	88.2 (83.3–94.1), 19	100.0 (81.7–100.0), 27	33.3 (16.7–57.1), 25	10.2 (−2.0–15.0), 29	50.0 (0.0–100.0), 19
	45–55	–	89.4 (85.7–95.0), 26	100.0 (100.0–100.0), 33	53.3 (28.6–118.0), 29	21.7 (−6.7–38.6), 31	70.0 (50.0–100.0), 21
	55–65	–	88.9 (82.1–93.1), 39	100.0 (56.2–100.0), 46	88.1 (20.8–150.0), 18	15.2 (0.4–34.2), 62	50.0 (50.0–100.0), 44
	> 65	–	91.7 (86.8–94.8), 21	100.0 (50.0–100.0), 24	68.6 (40.7–110.0), 18	5.6 (−3.9–17.1), 27	50.0 (0.0–100.0), 23
BMI	< 25	–	90.3 (85.5–94.8), 52	100.0 (80.0–100.0), 57	55.2 (25.0–110.0), 40	12.3 (−2.1–26.0), 68	87.5 (37.5–100.0), 52
	25–30	–	89.1 (82.7–92.9), 24	83.3 (50.0–100.0), 23	25.0 (3.6–62.5), 18	10.7 (0.0–44.8), 34	70.0 (50.0–100.0), 29
	> 30	–	88.2 (85.7–93.4), 29	100.0 (85.0–100.0), 50	68.1 (40.0–112.0), 32	12.7 (−2.3–21.5), 47	50.0 (0.0–75.0), 26
NP	Yes	–	89.9 (85.9–93.7), 62	100.0 (100.0–100.0), 73	50.0 (23.2–114.0), 51	12.7 (2.1–22.4), 91	87.5 (50.0–100.0), 60
	No	–	88.9 (83.5–94.6), 42	100.0 (50.0–100.0), 57	57.1 (26.8–100.0), 39	10.4 (−9.9–26.8), 57	50.0 (0.0–95.0), 46
GERD	Yes	–	91.3 (86.7–94.6), 66	100.0 (92.5–100.0), 86	55.2 (35.7–120.0), 54	11.1 (−2.3–28.6), 101	70.0 (50.0–100.0), 67
	No	–	85.7 (78.9–92.0), 38	100.0 (50.0–100.0), 44	40.8 (17.6–100.0), 36	14.5 (1.9–23.1), 47	50.0 (0.0–100.0), 39
Mepolizumab administration method	LYO	< 45	86.5 (70.6–88.2), 9	90.0 (76.2–100.0), 10	17.9 (3.3–44.6), 10	7.5 (−4.8–16.4), 12	0.0 (0.0–25.0), 7
	LYO	45–55	90.0 (85.1–95.6), 10	87.5 (25.0–100.0), 12	45.0 (19.5–104.0), 10	21.7 (−3.1–46.4), 11	50.0 (0.0–50.0), 8
	LYO	55–65	88.2 (81.5–93.5), 16	100.0 (50.0–100.0), 16	83.3 (9.5–121.0), 7	13.8 (0.0–25.0), 25	50.0 (0.0–50.0), 17

	LYO	> 65	89.7 (86.4–91.7), 9	100.0 (31.2–100.0), 10	68.1 (38.9–100.0), 8	0.6 (−6.6–7.2), 10	25.0 (0.0–68.8), 10
	AI/PFS	< 45	89.7 (89.7–92.2), 5	100.0 (100.0–100.0), 8	42.9 (26.2–53.6), 7	9.4 (−1.3–13.8), 8	100.0 (100.0–100.0), 6
	AI/PFS	45–55	90.8 (88.0–94.6), 7	100.0 (100.0–100.0), 9	56.7 (41.3–95.0), 8	19.7 (7.9–40.7), 9	100.0 (75.0–100.0), 5
	AI/PFS	55–65	88.3 (82.7–91.7), 12	100.0 (68.8–100.0), 16	84.0 (33.3–111.0), 6	16.9 (7.6–31.8), 19	87.5 (50.0–100.0), 14
	AI/PFS	> 65	91.4 (90.3–93.9), 6	100.0 (100.0–100.0), 7	110.0 (42.9–150.0), 5	17.1 (7.1–26.1), 8	100.0 (50.0–100.0), 7
	Homecare	< 45	94.1 (84.6–94.1), 5	100.0 (100.0–100.0), 9	46.4 (29.8–70.0), 8	10.4 (0.1–15.0), 9	70.0 (10.0–100.0), 6
	Homecare	45–55	89.2 (85.7–93.4), 9	100.0 (100.0–100.0), 12	53.3 (37.5–119.0), 11	21.7 (−18.2–33.5), 11	100.0 (72.5–100.0), 8
	Homecare	55–65	92.3 (81.2–93.4), 11	100.0 (75.0–100.0), 14	150.0 (100.0–178.0), 5	14.3 (2.1–38.0), 18	100.0 (75.0–100.0), 13
	Homecare	> 65	94.4 (92.7–96.7), 6	75.0 (58.3–100.0), 7	57.1 (50.0–110.0), 5	2.2 (−4.7–13.0), 9	75.0 (12.5–100.0), 6
Mepolizumab administration method/BMI	LYO	< 25	88.9 (69.7–95.4), 21	100.0 (50.0–100.0), 21	42.9 (20.5–90.0), 14	10.3 (−3.1–27.9), 25	50.0 (0.0–50.0), 20
	LYO	25–30	87.5 (80.6–93.8), 12	79.2 (43.8–100.0), 8	15.5 (0.0–51.8), 8	2.0 (−5.3–32.6), 15	50.0 (0.0–55.0), 12
	LYO	> 30	87.8 (85.9–91.0), 11	100.0 (50.0–100.0), 19	56.2 (40.0–83.3), 13	13.6 (−1.7–20.7), 18	0.0 (0.0–50.0), 10
	AI/PFS	< 25	91.0 (87.3–94.7), 15	100.0 (100.0–100.0), 19	58.6 (29.5–103.0), 14	15.7 (4.4–24.5), 22	100.0 (75.0–100.0), 17
	AI/PFS	25–30	89.7 (84.9–90.8), 6	100.0 (62.5–100.0), 7	35.7 (14.6–100.0), 4	23.8 (9.5–43.6), 9	100.0 (68.8–100.0), 8
	AI/PFS	> 30	89.7 (86.7–92.2), 9	100.0 (100.0–100.0), 14	63.3 (40.5–101.0), 8	13.9 (0.0–21.3), 13	50.0 (50.0–75.0), 7
	Homecare	< 25	93.1 (88.5–94.7), 16	100.0 (80.0–100.0), 17	55.2 (45.4–112.0), 12	10.8 (−14.3–24.6), 21	100.0 (75.0–100.0), 15
	Homecare	25–30	88.1 (80.2–93.0), 6	87.5 (62.5–100.0), 8	31.0 (23.2–58.3), 6	27.3 (1.5–44.2), 10	100.0 (50.0–100.0), 9
	Homecare	> 30	91.8 (83.3–94.1), 9	100.0 (100.0–100.0), 17	80.0 (46.4–134.0), 11	10.3 (−2.6–17.3), 16	75.0 (0.0–75.0), 9
Mepolizumab administration method/NP	LYO	Yes	88.0 (84.5–92.6), 24	100.0 (76.2–100.0), 26	37.9 (21.4–88.8), 18	10.3 (0.0–22.4), 33	50.0 (0.0–50.0), 22
	LYO	No	88.9 (71.0–94.4), 19	62.5 (25.0–100.0), 22	56.2 (13.3–83.3), 17	9.7 (−4.4–25.7), 24	0.0 (0.0–50.0), 19

	AI/PFS	Yes	90.9 (89.7–93.1), 18	100.0 (100.0–100.0), 22	46.7 (23.2–81.7), 15	14.8 (7.9–22.0), 29	100.0 (62.5–100.0), 19
	AI/PFS	No	86.3 (83.7–92.5), 12	100.0 (78.8–100.0), 18	80.0 (45.2–125.0), 11	19.7 (−7.0–27.7), 15	75.0 (50.0–100.0), 13
	Homecare	Yes	92.1 (85.1–93.9), 20	100.0 (100.0–100.0), 25	61.9 (44.6–120.0), 18	12.7 (2.2–29.4), 29	100.0 (62.5–100.0), 19
	Homecare	No	94.0 (85.2–95.2), 11	100.0 (80.0–100.0), 17	46.7 (33.3–95.0), 11	–1.2 (−15.8–26.2), 18	75.0 (50.0–100.0), 14
Mepolizumab administration method/GERD	LYO	Yes	89.5 (86.4–94.9), 26	100.0 (95.0–100.0), 28	50.0 (30.4–89.6), 18	6.4 (−5.6–24.4), 38	50.0 (0.0–50.0), 24
	LYO	No	85.7 (69.7–92.9), 17	50.0 (12.5–87.5), 20	33.3 (14.3–80.0), 17	13.8 (1.0–22.1), 19	0.0 (0.0–50.0), 17
	AI/PFS	Yes	91.4 (89.2–93.7), 20	100.0 (100.0–100.0), 29	55.2 (42.9–111.0), 18	13.9 (3.3–31.7), 31	100.0 (50.0–100.0), 22
	AI/PFS	No	87.3 (83.5–89.9), 10	100.0 (100.0–100.0), 11	39.5 (17.1–90.0), 8	16.7 (8.8–23.8), 13	100.0 (56.2–100.0), 10
	Homecare	Yes	93.9 (88.5–95.0), 20	100.0 (80.0–100.0), 29	61.9 (44.6–142.0), 18	10.3 (−2.9–29.7), 32	100.0 (50.0–100.0), 21
	Homecare	No	85.7 (81.3–92.3), 11	100.0 (100.0–100.0), 13	46.7 (23.8–105.0), 11	13.0 (0.0–23.2), 15	100.0 (37.5–100.0), 12

**Quantity**	**Group**	**Subgroup**	**The rate of improvement of a given quantity (6–9 vs. 0 months) (%)**				
			**BEC**	**ER**	**ACT**	**FEV1**	**OCS**
Mepolizumab administration method	LYO	–	91.4 (85.7–97.8), 38	95.0 (50.0–100.0), 44	45.2 (18.7–80.0), 32	11.3 (−3.7–26.7), 52	50.0 (31.2–100.0), 38
	AI/PFS	–	90.8 (86.3–95.8), 28	100.0 (100.0–100.0), 30	65.5 (31.2–120.0), 20	12.5 (1.7–23.8), 36	100.0 (50.0–100.0), 26
	Homecare	–	92.3 (86.3–95.8), 32	100.0 (97.5–100.0), 40	62.5 (32.4–112.0), 28	13.9 (2.0–26.5), 42	100.0 (75.0–100.0), 33
Age (years)	< 45	–	91.8 (85.3–96.6), 22	100.0 (75.0–100.0), 25	46.4 (24.8–70.4), 24	7.6 (0.5–18.5), 30	77.5 (21.2–100.0), 18
	45–55	–	93.3 (86.7–97.6), 26	100.0 (78.8–100.0), 28	40.8 (19.5–102.0), 26	15.1 (−5.0–37.0), 31	80.0 (50.0–100.0), 21
	55–65	–	89.7 (85.7–94.9), 33	100.0 (90.0–100.0), 41	93.3 (33.0–178.0), 16	13.1 (0.8–24.6), 47	100.0 (50.0–100.0), 40
	> 65	–	89.7 (86.4–97.8), 17	100.0 (75.0–100.0), 20	70.0 (47.1–119.0), 14	14.9 (−1.5–26.8), 22	100.0 (50.0–100.0), 18
BMI	< 25	–	91.4 (85.7–95.4), 50	100.0 (90.0–100.0), 53	51.7 (20.5–112.0), 38	13.3 (0.0–26.0), 59	100.0 (50.0–100.0), 48
	25–30	–	89.6 (83.3–96.5), 21	100.0 (50.0–100.0), 21	42.9 (8.6–79.2), 14	21.9 (−0.1–41.4), 27	90.0 (50.0–100.0), 26

	> 30	–	93.9 (87.8–96.8), 27	100.0 (75.0–100.0), 40	68.3 (42.1–120.0), 28	10.1 (−8.4–20.5), 44	50.0 (50.0–78.5), 23
NP	Yes	–	93.2 (89.4–97.9), 54	100.0 (100.0–100.0), 65	57.7 (32.1–120.0), 46	13.2 (3.6–26.4), 76	100.0 (50.0–100.0), 55
	No	–	88.1 (83.7–94.9), 44	90.0 (50.0–100.0), 49	46.9 (19.2–80.0), 34	8.7 (−12.6–24.0), 54	62.5 (50.0–100.0), 42
GERD	Yes	–	93.6 (89.5–97.1), 60	100.0 (90.0–100.0), 78	66.7 (42.9–120.0), 51	13.1 (−2.4–26.7), 91	80.0 (50.0–100.0), 63
	No	–	85.8 (80.6–92.7), 38	100.0 (50.0–100.0), 36	28.6 (6.7–66.7), 29	10.8 (0.0–23.8), 39	100.0 (50.0–100.0), 34
Mepolizumab administration method/Age (years)	LYO	< 45	94.4 (87.9–98.6), 9	83.3 (60.0–100.0), 9	42.9 (19.0–62.5), 9	4.1 (−3.6–15.4), 11	22.5 (5.0–62.5), 6
	LYO	45–55	92.8 (86.6–96.1), 10	73.3 (54.2–100.0), 10	25.0 (8.3–46.7), 9	10.9 (−3.9–36.2), 11	50.0 (50.0–81.2), 8
	LYO	55–65	88.9 (85.9–93.8), 11	100.0 (75.0–100.0), 17	66.7 (28.8–157.0), 8	14.0 (0.6–27.9), 20	62.5 (43.8–100.0), 16
	LYO	> 65	88.1 (82.6–98.0), 8	75.0 (50.0–100.0), 8	85.7 (47.1–119.0), 6	6.0 (−4.8–23.7), 10	75.0 (43.8–100.0), 8
	AI/PFS	< 45	87.5 (84.5–94.6), 6	100.0 (100.0–100.0), 7	64.3 (38.1–73.3), 7	2.3 (−2.1–17.1), 9	100.0 (40.0–100.0), 5
	AI/PFS	45–55	96.3 (93.5–98.7), 8	100.0 (100.0–100.0), 7	53.3 (21.3–135.0), 7	12.8 (6.2–40.7), 9	100.0 (80.0–100.0), 5
	AI/PFS	55–65	89.4 (82.0–91.3), 10	100.0 (100.0–100.0), 10	149.0 (134.0–163.0), 2	10.1 (1.7–21.0), 12	100.0 (50.0–100.0), 11
	AI/PFS	> 65	92.0 (88.6–95.5), 4	100.0 (100.0–100.0), 6	76.4 (37.5–112.0), 4	25.2 (18.5–28.0), 6	100.0 (50.0–100.0), 5
	Homecare	< 45	89.7 (84.9–95.6), 7	100.0 (100.0–100.0), 9	56.4 (29.8–74.4), 8	12.7 (9.4–21.7), 10	100.0 (60.0–100.0), 7
	Homecare	45–55	91.2 (84.8–94.5), 8	100.0 (100.0–100.0), 11	47.5 (32.1–98.5), 10	15.2 (−9.7–27.6), 11	100.0 (78.8–100.0), 8
	Homecare	55–65	92.6 (88.0–95.3), 12	100.0 (60.0–100.0), 14	104.0 (27.1–171.0), 6	14.6 (3.5–30.9), 15	100.0 (50.0–100.0), 13
	Homecare	> 65	92.3 (89.6–97.3), 5	100.0 (81.2–100.0), 6	70.0 (66.8–90.0), 4	8.2 (3.2–22.8), 6	100.0 (100.0–100.0), 5
Mepolizumab administration method/BMI	LYO	< 25	90.5 (85.3–96.6), 19	95.0 (65.0–100.0), 20	42.9 (13.0–64.6), 15	12.5 (−2.8–26.7), 24	50.0 (50.0–100.0), 18
	LYO	25–30	91.8 (80.6–98.2), 8	75.0 (50.0–100.0), 9	61.9 (9.9–102.0), 6	10.8 (0.9–47.8), 12	75.0 (37.5–100.0), 11
	LYO	> 30	93.8 (87.1–95.6), 11	100.0 (50.0–100.0), 15	43.8 (39.2–100.0), 11	10.3 (−11.5–20.5), 16	50.0 (25.0–75.0), 9

	AI/PFS	< 25	90.9 (86.3–96.2), 16	100.0 (100.0–100.0), 15	110.0 (33.9–120.0), 11	16.7 (5.2–24.1), 17	100.0 (100.0–100.0), 14
	AI/PFS	25–30	88.0 (84.5–89.7), 6	100.0 (100.0–100.0), 6	21.4 (19.0–44.0), 3	5.6 (−4.2–29.7), 7	100.0 (65.0–100.0), 7
	AI/PFS	> 30	94.7 (92.3–96.4), 6	100.0 (100.0–100.0), 9	66.7 (45.5–132.0), 6	10.1 (−4.2–19.3), 12	50.0 (50.0–50.0), 5
	Homecare	< 25	92.3 (82.7–94.1), 15	100.0 (100.0–100.0), 18	55.2 (37.4–83.6), 12	12.7 (5.9–24.6), 18	100.0 (100.0–100.0), 16
	Homecare	25–30	89.6 (84.9–91.3), 7	100.0 (62.5–100.0), 6	28.6 (5.9–83.3), 5	23.6 (16.9–43.2), 8	100.0 (78.8–100.0), 8
	Homecare	> 30	95.6 (92.2–97.5), 10	100.0 (75.0–100.0), 16	70.0 (54.8–115.0), 11	9.9 (−1.7–24.9), 16	75.0 (50.0–80.0), 9
Mepolizumab administration method/NP	LYO	Yes	93.9 (88.3–97.9), 18	100.0 (100.0–100.0), 23	46.7 (25.0–80.0), 17	13.9 (2.2–26.6), 29	62.5 (50.0–100.0), 20
	LYO	No	87.7 (84.8–95.3), 20	66.7 (50.0–83.3), 21	43.8 (7.5–73.3), 15	0.0 (−11.6–26.2), 23	50.0 (25.0–93.8), 18
	AI/PFS	Yes	92.3 (89.6–98.6), 17	100.0 (100.0–100.0), 19	48.1 (24.1–120.0), 12	12.5 (5.3–22.8), 22	100.0 (87.5–100.0), 16
	AI/PFS	No	88.5 (84.8–95.2), 11	100.0 (95.0–100.0), 11	95.0 (56.5–114.0), 8	13.0 (−5.5–24.0), 14	65.0 (50.0–100.0), 10
	Homecare	Yes	92.3 (89.6–97.1), 19	100.0 (100.0–100.0), 23	70.0 (53.3–120.0), 17	14.6 (7.0–30.7), 25	100.0 (90.0–100.0), 19
	Homecare	No	87.8 (81.5–93.3), 13	100.0 (75.0–100.0), 17	40.0 (22.6–70.0), 11	12.2 (−17.1–23.5), 17	78.5 (50.0–100.0), 14
Mepolizumab administration method/GERD	LYO	Yes	93.5 (89.5–97.9), 22	100.0 (75.0–100.0), 29	66.7 (42.9–135.0), 19	14.1 (−5.3–30.0), 35	50.0 (50.0–100.0), 24
	LYO	No	86.1 (80.6–96.0), 16	50.0 (37.5–100.0), 15	8.3 (−5.9–43.8), 13	1.7 (−3.5–15.0), 17	62.5 (25.0–100.0), 14
	AI/PFS	Yes	93.1 (89.3–97.0), 18	100.0 (100.0–100.0), 23	64.3 (38.1–115.0), 15	11.7 (−2.4–23.7), 28	100.0 (50.0–100.0), 19
	AI/PFS	No	86.1 (81.4–93.3), 10	100.0 (100.0–100.0), 7	112.0 (19.0–120.0), 5	16.9 (5.5–27.0), 8	100.0 (75.0–100.0), 7
	Homecare	Yes	94.1 (89.7–96.6), 20	100.0 (92.5–100.0), 26	70.0 (53.3–150.0), 17	14.8 (1.4–30.9), 28	100.0 (68.8–100.0), 20
	Homecare	No	84.6 (79.0–92.3), 12	100.0 (100.0–100.0), 14	40.0 (23.8–64.2), 11	12.7 (5.9–23.0), 14	100.0 (75.0–100.0), 13

Abbreviations: ACT, asthma control test; AI, autoinjectors; BEC, blood eosinophil count; BMI, body mass index; ER, exacerbation rate; FEV1, forced expiratory volume; GERD, gastroesophageal reflux; IQR, interquartile range; LYO, lyophilised formulation; NP, nasal polyposis; OCS, oral corticosteroids; PFS, prefilled syringes
